# Causal Discovery in Sepsis—A (Random) Walk in the Park?

**DOI:** 10.1001/jamanetworkopen.2025.27496

**Published:** 2025-08-01

**Authors:** Andrew J. Admon, Erin F. Carlton

**Affiliations:** Division of Pulmonary and Critical Care Medicine, Department of Internal Medicine, University of Michigan, Ann Arbor; Department of Epidemiology, University of Michigan, Ann Arbor; Veterans Affairs (VA) Center for Clinical Management Research, VA Ann Arbor Healthcare System, Ann Arbor, Michigan; Division of Critical Care Medicine, Department of Pediatrics, University of Michigan, Ann Arbor; Susan B. Meister Child Health Evaluation and Research Center, University of Michigan, Ann Arbor

Sepsis is acute organ failure resulting from a dysregulated host response to infection.^1^ Given the marked patient-level heterogeneity in sepsis onset, clinical course, and recovery, investigators have long sought to identify characteristics that predispose patients to sepsis or its severe manifestations. The goals of these investigations are often to generate mechanistic insights that might predict observed heterogeneity or enable novel therapies (ie, treatable traits). In this issue of *JAMA Network Open*, Sriram and colleagues^2^ evaluated the role of Epstein-Barr virus (EBV) in sepsis-related immune dysregulation. EBV is a highly prevalent virus implicated in several autoimmune diseases and hematologic malignant neoplasms. Prior work demonstrated an association between EBV seropositivity and mortality among children hospitalized with sepsis.^3^ In the current study, the authors evaluated whether specific biomarkers that reflect inflammation, immune system dysregulation, and thrombotic microangiopathy mediate the observed association between EBV positivity and mortality. Ultimately, Sriram et al^2^ found that EBV seropositivity was associated with several biomarkers, many of which are known proinflammatory cytokines or markers of acute inflammation. In a subsequent analysis using a causal discovery algorithm, the authors identified potential pathways through which EBV positivity may influence mortality. Together, these findings support the hypothesis that EBV seropositivity may contribute to greater mortality in sepsis by promoting acute immune dysregulation.

Other studies in children and adults with sepsis have sought to unpack the heterogeneity of sepsis biology as well as classify individuals who are at risk for poorer outcomes. Indeed, several phenotypes (groups based on observable characteristics) or endotypes (subclasses based on molecular biology) have been identified, described, and validated. These groups may help to explain differences in outcomes among patients with sepsis. Most relevant to the current study, the Phenotyping Pediatric Sepsis-Induced Multiple Organ Failure (PHENOMS) study from the Eunice Kennedy Shriver National Institutes of Child Health and Human Development Collaborative Pediatric Critical Care Research Network described 3 inflammatory phenotypes. These phenotypes—immunoparalysis associated multiple organ failure (MOF), sequential liver failure associated MOF, and thrombocytopenia associated MOF—occurred in 25% of children and were associated with both macrophage activation syndrome and mortality.^4^ In a secondary analysis of this cohort, EBV seropositivity was associated with mortality.^3^ However, it remained unclear whether EBV seropositivity was merely associated with other factors that led to severe illness (eg, via a common prior cause such as exposure history or susceptibility to infection) or was a mechanistic part of the underlying pathway between sepsis onset and death.

To address this gap, Sriram and colleagues^2^ used modern computational techniques for identifying potential mechanistic relationships among variables in observed data. These algorithmic “causal discovery” approaches begin with large datasets that contained many variables.^5^ They infer potential relationships among those variables by examining statistical associations between pairs of variables while controlling for others. This process identifies a network of statistically associated variables, often represented graphically as nodes (variables) connected by edges (associations).

Sriram et al^2^ then applied another algorithm to convert this undirected (ie, arrowless) network into a directed graph that represented potential cause-and-effect relationships. Many such algorithms infer directionality by leveraging temporal ordering, prior knowledge, and the principle that statistical associations behave differently depending on how they are connected (eg, conditioning on a common cause often eliminates a statistical association, whereas conditioning on a common effect often creates one). The result is a directed graph showing the potential causal associations among variables, beginning with EBV seropositivity in this study and ending with death.

Finally, the authors use a random walk algorithm to identify the strongest pathways through this directed graph. Starting at EBV positivity, the algorithm traverses the network with step probabilities proportional to the strengths of associations among variables. By pruning out pathways that are uncommon, inconsistent, or less direct, the algorithm produces a simplified model that reflects the strongest (ie, most supported by observations in the dataset) mechanistic pathways of the association of EBV seropositivity and death.

The strengths of this approach rest in its ability to leverage high-dimensional data and prior observations (such as the prior association between EBV seropositivity and death in pediatric sepsis) to identify plausible biological mechanisms worthy of further study. However, as with other approaches for identifying potential causal relationships in observational datasets, causal discovery algorithms are most accurate when supported by subject matter expertise to set starting parameters and restrict the set of potential relationships to those that are plausible and consistent with prior work.^5^ Moreover, the resulting causal diagram is highly dependent on the set of available input variables, the granularity with which intermediate pathways are identified and measured, and any unmeasured confounding or selection bias that may be influencing the observations in the dataset. These are all design features of the parent study that require prior insight and deliberation. In the current study by Sriram et al,^2^ the timing of measurement also matters; immune response in sepsis is dynamic, and biomarker measurement at baseline might not reflect dominant pathways at other points during illness and recovery, including key intervention points. Finally, the risks of overfitting (that model output reflects nuances of this study population rather than broader truths among children with sepsis) and model instability (given the small source population, particularly among some subgroups) warrant further validation of these findings in other cohorts.

Despite these limitations, the authors found that EBV seropositivity (ie, latent EBV infection) may have a plausible mechanistic relationship with mortality among children with sepsis through immune dysregulation. Specifically, EBV seropositivity had both a direct effect on mortality (likely via other, unmeasured mediators) and an indirect effect via hyperferritinemia and macrophage activation syndrome. These findings, in alignment with the group’s prior work,^3^ support the vital role of immune dysregulation in sepsis outcomes and suggest EBV positivity as an upstream contributing cause.^6^

These findings are notable for 2 reasons. First, EBV seropositivity can be measured, as done in this study, via viral capsid antigen IgG—a test that is commonly available to bedside clinicians, unlike many other specialized sepsis-related biomarkers. Second, there are several plausible ways by which EBV-induced acute immune dysregulation could potentially be mitigated, including targeting the AMP-activated protein kinase pathway (through medications such as metformin) or mitigation of the EBV-hyperferritinemia pathway.^6–8^ Although the implementation of many of these treatment strategies are still in their early or experimental stages (and further validation of the findings by Sriram et al^2^ is necessary), the Collaborative Pediatric Critical Care Research Network is currently conducting the ongoing Targeted Reversal of Inflammation in Pediatric Sepsis-Induced Multiple Organ Dysfunction (TRIPS) study, a randomized clinical trial of anakinra vs placebo for hyperferritenimic sepsis.^9^ Although the TRIPS study does not enrich the study population for children with EBV, it may prove informative, given the results shared by Sriram et al,^2^ to evaluate treatment effects among members of this subgroup.

This study by Sriram et al^2^ exemplifies how modern computational causal methods can support our understanding of complex diseases. Beyond pediatric sepsis, such approaches offer promising avenues for leveraging rich, multiomic datasets to understand mechanistic pathways, unpack heterogeneity, and identify actionable targets (eg, treatable traits) for novel precision therapeutics. When results are properly validated and algorithms are combined with expert involvement from study design and data collection to model interpretation, such causal discovery methods may prove instrumental in transforming our approach to understanding and caring for human diseases.
